# Ultrasound Elastography in Temporomandibular Disorders: A Narrative Review

**DOI:** 10.7759/cureus.70004

**Published:** 2024-09-23

**Authors:** Deepa J Patil, Rajesh K Rathore, Ashutosh Patel

**Affiliations:** 1 Oral Medicine and Radiology, K.M. Shah Dental College and Hospital, Sumandeep Vidyapeeth (Deemed to Be University), Vadodara, IND; 2 Radiology, Smt. B.K. Shah Medical Institute and Research Centre, Sumandeep Vidyapeeth (Deemed to Be University), Vadodara, IND

**Keywords:** masseter, shear wave elastography, strain wave elastography, temporomandibular disorders, ultrasound elastography

## Abstract

A class of intricate musculoskeletal diseases known as temporomandibular disorders (TMDs) affects the temporomandibular joint (TMJ) and its supporting structures. The majority of individuals will at some point in their lives experience some degree of TMD symptoms, as these diseases are highly prevalent in the general population. TMDs are multifactorial and are attributed to various physical and biopsychosocial factors. The TMD patients typically experience preauricular pain, tenderness of masticatory muscles, and joint sounds, and these in turn affect their quality of life. To carry out the appropriate course of treatment, it is critical to make an accurate and timely diagnosis. The TMDs are classified as myofascial pain, internal disc derangement, and degenerative disorders of TMJ. Myofascial pain, which is identified by palpating the affected muscles of mastication and tenderness, is one of the most common findings. The muscles in this condition become stiff due to the contraction of myofibrils and are known as trigger bands. The diagnosis of trigger bands involving the masticatory muscles commonly involving the masseter muscle in myofascial pain to date is subjective, and palpation is the only tool used for its diagnosis. An objective assessment of the masticatory muscles is desirable for accurate diagnosis and treatment planning. Various tools like electromyography and hardness meters have been for assessing muscle stiffness, but their application in TMJ muscle disorders has not yielded valuable results. A novel diagnostic method called ultrasound elastography evaluates muscle stiffness both qualitatively and quantitatively using an elastogram and the muscular elasticity index. In this paper, we will review the ultrasound elastographic techniques utilized for the diagnosis and management of TMDs.

## Introduction and background

According to Schiffman and Ohrbach [1], temporomandibular disorders (TMDs) are a group of complex musculoskeletal conditions affecting the temporomandibular joint (TMJ) and associated structures. They have proposed diagnostic criteria for temporomandibular disorders (DC-TMD) which classify into masticatory, disc derangement, and degenerative disorders of the joint. These disorders can lead to pain, dysfunction, and an impaired quality of life. Accurate diagnosis and assessment of these conditions are crucial for effective management. The epidemiology of TMDs suggests a high prevalence in the adult population, with estimates ranging from 50% to 80%. These disorders are characterized by a multifactorial etiology, with biopsychosocial factors playing a significant role. According to Pitta et al. [2], the diagnostic process can be complicated due to the involvement of various structures and the complex clinical presentation.

Ferrario et al. [3] have emphasized that musculoskeletal changes, such as alterations in the mechanical properties of the orofacial and neck muscles, have been reported in patients with TMDs. Many tools have been used to assess tissue stiffness. Surface electromyographic analysis during clenching has been used to differentiate patients with TMJ disorders from those with neck pain problems. However, according to Suvinen et al. [4], the clinical value of electromyographic assessment has been questioned due to variable and controversial findings, the lack of normative data, and methodological or reliability issues.

Ophir et al. [5] first described ultrasound elastography (USE) as a relatively new non-invasive imaging technique that has the potential to provide valuable information about the mechanical properties of the tissues involved in TMDs. Shiina et al. [6] have described the various ultrasound-based elastography techniques, and they can be classified as either static, like strain elastography (SE), or dynamic, like shear wave elastography (ShWE). The primary difference between these methods is how the mechanical stimuli are applied to the tissue of interest. Furthermore, in the application, there is a variation among these techniques based on the degree to which the transducer provides mechanical energy to the tissues. USE may play a crucial role in the diagnosis process, assessment, and better understanding of the pathophysiology of the TMJ. This method allows an enhanced knowledge of the pathological changes that are present in the TMJ. Olchowy et al. [7] in their systematic review emphasized that ultrasound elastography is a promising method for evaluating masseter muscle elasticity and further investigations on larger cohorts are necessary to ascertain the precision of elastography in characterizing masticatory muscle diseases.

Rationale

According to Peck et al. [8], TMDs have a multifactorial etiology and are a group of musculoskeletal and neuromuscular conditions that involve the masticatory muscles, the TMJ, and associated structures, and impair the ability to perform typical daily functions, proper nutrition, and good mental and psychological status. These disorders are very common in the general population, with most individuals experiencing some level of TMD symptoms during their lifetime. It is important to be able to make an accurate and early diagnosis to perform proper treatment. Therefore, it is of great relevance for both the patient and the attending physician.

After the patient's history and examination, imaging plays a key role in the diagnosis of TMD. Talmaceanu et al. [9] have described the use of modern imaging techniques, such as magnetic resonance imaging (MRI) or ultrasound, which has significantly improved the diagnosis of these disorders, allowing for the study of joint anatomy as well as the structures involved, increasing the accuracy of disorders detection and the success of therapeutic interventions. In recent years, USE has been increasingly used and embodied in the daily practice of many aspects of medicine, where changes in tissue stiffness are present, such as breast, liver, and prostate areas.

According to Dworkin et al. [10], only 15% of the general population exhibits TMD symptoms, despite the fact that 60% to 70% of them have TMDs. According to a study by Poluha et al. [11], one of the most frequent findings in TMD is myofascial pain, which is diagnosed by palpating the area and feeling pain and tenderness. Regional discomfort at sensitive spots, referred to as trigger bands, which manifest as tense bands of skeletal muscle and tendon, is frequently linked to myofascial pain and is due to the contraction of myofibrils. In clinical terms, palpation reveals a tight, stiff masticatory muscle linked to myofascial pain.

According to Yu et al. [12], it is challenging to evaluate the disease scientifically because the diagnosis is based on a subjective evaluation made by patients and medical professionals. Nonetheless, this is a judgment made by skilled medical professionals with extensive training. It is currently unknown how well masticatory muscle stiffness in myofascial pain patients may be objectively assessed. As a result, a reliable technique for assessing the degree of masticatory muscle stiffness and the impact of therapy on myofascial pain must be established.

Numerous instruments, such as myotonometry and muscle hardness meters, have been employed to measure the stiffness in myofascial pain, but their application to TMDs is limited. While EMG has been used to assess changes in the masseter muscles, this technique has drawbacks because the experimental environment can readily modify the electrical signal [13].

Kamaya et al. [14] have described ultrasonography as an alternative modality that can noninvasively display the structure and thickness of the masseter and other muscles that are located superficial to the bone structures in the head and neck area. The benefit of conventional ultrasonography of being a cheap, adaptable, and extensively accessible modality that can be applied by the patient's bed is extended to ultrasonography elastography (USE) as well. USE has been investigated for a number of clinical uses in recent times and has been implemented into clinical practice for particular applications like the evaluation of liver fibrosis or breast-characterizing lesions. USE's elasticity imaging offers supplementary data to traditional ultrasonography by including rigidity as an additional quantifiable characteristic of modern ultrasonography imaging methods.

## Review

Principles of ultrasound elastography

Alteration in tissue stiffness has been linked to a number of disorders, including calcification, atheroma, fibrosis linked to liver cirrhosis, and malignant tumors. Advanced imaging modalities like CT, MRI, and PET have been used for morphological diagnostic imaging and function. Determining tissue stiffness objectively has been possible with the advent of USE. Measuring the stiffness of tissue will help in the assessment of early detection and diagnosis reflecting the qualitative changes when morphological alterations are not evident. It can also be utilized to assess response to treatment and improve the accuracy of diagnosing diseases with fibrosis [15].

According to Garra [16], elastography helps to differentiate the biomechanical characteristics of healthy from diseased tissues. Traditionally, palpation has been used to assess the hardness or stiffness of the tissue. Elastography is an imaging modality that is an extension of palpation. Elastography is a non-invasive technique for measuring strain and the distribution of elastic modulus in soft tissues [17].

Sigrist et al. have described [18] the basic workflow for elastography as follows: (1) use a quasi-static, harmonic, or transient mechanical source to perturb the tissue; (2) measure the resulting mechanical response (displacement, strain, or vibration's amplitude and phase); and (3) use the measured mechanical response to infer the biomechanical properties of the underlying tissue by using a simplified or continuum mechanical model.

Gennisson et al. [19] in their review have emphasized that the initial work on elastography started in the 1980s in England. The attempts to standardize the B mode images failed due to the lack of standard uniform force leading to variations in the resultant image. Subsequently, Doppler imaging was used to generate the images, and the term was known as sonoelastography. Ophir et al. in the year 1991 recognized the strain wave elastographic technique, which is presently in use in all advanced imaging centers. The next development was the advent of ShWE, which is being widely used worldwide nowadays.

The explanation of elasticity, in simple words, is the characteristic of solid material to revert back to its initial form and dimensions on the cessation of external pressure. The concept of elasticity is based on the principles of Hooke's law, which is traditionally defined as the proportionality of stress to strain. Define stress as the deforming force exerted on matter and strain as the change in dimensions (size) of the substance. In mathematical terminology, Hooke's law is formulated as: \[F = k \Delta x\] where the force applied per unit area or stress, denoted as F, is measured in newtons per square meter or pascals; k is the constant of proportionality that is one unit smaller; Δx is the spatial dimension change (size), measured in centimeters [20].

The atoms and molecules that make up an object have a property called elastic modulus, which is independent of the object's size or shape. To put it simply, the elastic moduli of two blocks of the same shape and size, one made of wood and the other of iron, will differ. Depending on the way the deforming pressures are directed, three categories of elastic moduli are identified. These three kinds are used in various elastography applications. Young's modulus, the kind of modulus used in strain elastography, has a force perpendicular to the object's opposing faces. The universally applicable modulus for all types of dynamic elastography is the in-shear modulus, which is determined by the force acting tangentially to one face of the object. The force in bulk modulus is oriented at a right angle to all the faces of the object, therefore crucial for speckle tracking, which forms the basis of all elastography techniques [21].

There are many USE methods accessible depending on the force application and data processing mechanism. Strain wave and ShWE are the predominant techniques with which USE is conducted.

Strain wave elastography

According to Barr et al. [22], Dr. Jonathan Ophir first created strain imaging in the 1970s and called it elastography. After his pivotal research, a number of studies for measuring strain and strain elastography (StWE) were commercialized. This was the initial form of elastography adopted for the practical measurement of tissue deformation caused by the application of pressure with a probe on the surface of the body. Its application entails the manual application of pressure, similar to palpation, or via the use of cardiovascular pulsation. This technology is being employed in several medical specialties, such as the detection of malignancy of the breast, liver fibrosis, and musculoskeletal imaging.

**Figure 1:**
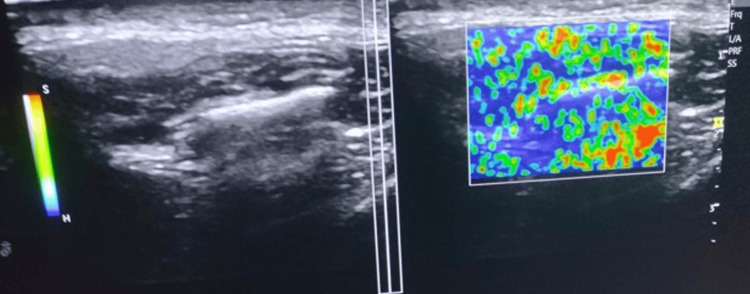
Color-coded elastogram generated by strain wave elastography

The principle of StWE is explained by the following formula: \[\epsilon = \frac{d\frac{\partial}{dz} \rightarrow \delta_2 - \delta_1}{L}\] By carefully applying gentle pressure to the tissue using a probe in the beam direction, we can determine the displacement d(z) at each site z by comparing the echo signal before and after compression. Next, the strain (ε), which represents the deformation ratio between two adjacent points with an interval, L, can be calculated using the above formula [23]. The mechanism of StWE, according to Klauser et al. [24], is that tissue compression results in displacement or strain. Stiffer tissue exhibits minimal strain, while softer tissue experiences greater strain. Tissue displacement is determined by performing repeated manual compression with a handheld ultrasound transducer. The displacement distribution is typically visualized in an elastogram, which is commonly presented as a color-coded image overlaid on the B-mode image. The use of different colors is common in medical imaging to represent varying levels of tissue elasticity. In this case, red is often used to represent softer tissue, while blue is used to depict harder tissue. Intermediate elasticity is typically depicted using yellow or green (Figure 1).

It is worth mentioning that the color reflects the varying stiffness of the tissues, allowing for potential adjustments to the color coding. The reliability of compression elastography poses a challenge as the level of compression can distort the elastogram, leading to nonlinear changes in tissue elasticity [25].

**Figure 2:**
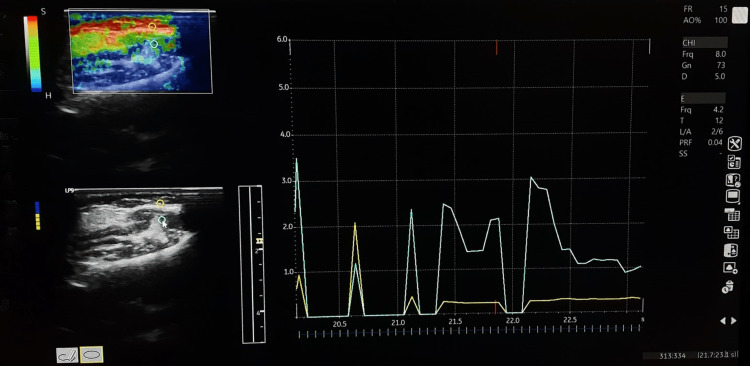
Graph depicting the muscle elasticity index generated by ratio of elasticity index of masseter muscle with the surrounding subcutaneous tissue

Most manufacturers provide the amount of manual compression (strain) as a visual indicator on the screen inside the area of interest (ROI), while a quality factor greater than 60 indicates the ideal compression force. The amount of stress applied is not quantifiable like elastic modulus [26]. Therefore, StWE is a qualitative measure of stiffness. However, certain systems offer semiquantitative techniques for analyzing image features like a strain ratio. This measures the target ROI's relative elasticity in comparison to the reference ROI, which is often the subcutaneous fat layer. This is termed the MEI (Figure 2).

The disadvantage of StWE is that the method is subjective and not reproducible. It will depend on the amount of stress applied by the transducer by the individual radiologist [27].

ShWE

According to Ličen and Kozinc [28], ShWE has become increasingly common in the assessment of different musculoskeletal tissues in research and clinical settings over the last decade. It enables the measurement of tissue elasticity both qualitatively and quantitatively. This technique is constantly advancing for new applications and clinical usefulness in musculoskeletal imaging. ShWE enhances the diagnosis obtained at gray-scale (B-mode) ultrasound and power and color Doppler ultrasound by quantifying mechanical and elastic tissue properties. The shear wave is a transverse wave that occurs in an elastic medium that is influenced by a periodic shear force. The concept of shear involves the alteration of a substance layer's shape without any accompanying change in volume. This alteration is brought about by a pair of equal forces that act in opposite directions along the two opposing sides of the layer. Following the shear interaction, the initial layer (tissue) will return to its original shape, while the neighboring layers experience shear and cause additional movement of the shear wave, which propagates as a transverse shear wave.

**Figure 3:**
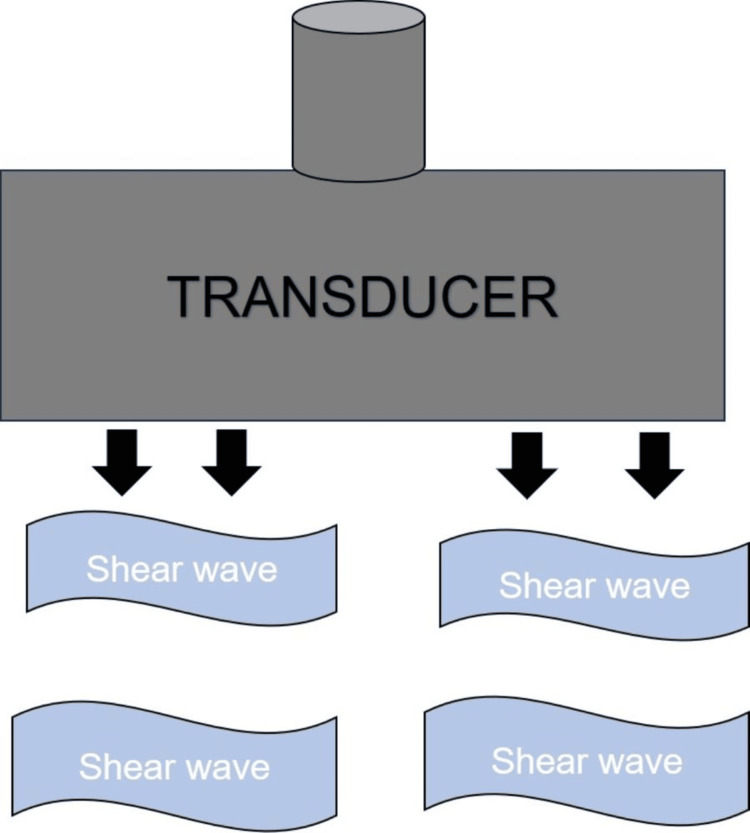
Pictorial description of generation of shear wave by the ultrasound transducer Image credits: Dr. Deepa J. Patil

Taljanovic et al. [29] have outlined the process of generating shear waves and determining the shear modulus. During the first step, shear waves are generated through the use of concentrated acoustic radiation force from a linear ultrasound array. This process creates local stress and induces displacement in the tissue (Figure 3).

**Figure 4:**
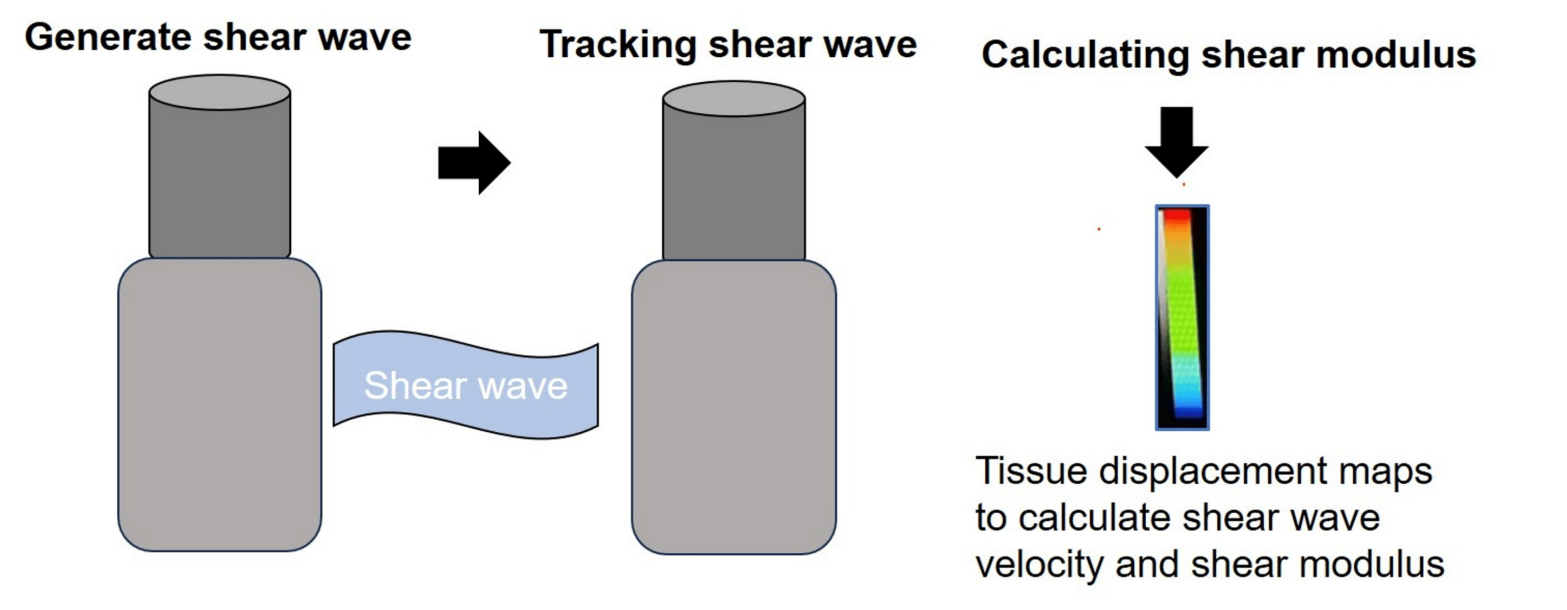
Step-wise description of shear wave generation and calculation of elastic modulus Image credits: Dr. Deepa J. Patil

Shear waves are produced and travel through the surrounding tissues in a transverse plane, perpendicular to the primary wave. These waves move at a slower velocity and result in displacements in the tissue. The second step involves utilizing fast plane wave excitation to monitor the movement of the tissue and measure the velocities of the shear waves as they travel. Tissue displacement is determined through the use of a speckle tracking algorithm. The third step utilizes tissue displacement maps to determine shear-wave velocity (cs), typically measured in meters per second (Figure 4).

The shear modulus calculated by dividing stress by strain is expressed as: \[G = rcs^2\]

where r is the numerical measurement that can be expressed as the speed of shear waves in meters per second (m/sec) or as the modulus for calculating tissue elasticity based on shear in kilopascals (kPa) [29].

On the ultrasound screen, quantitative shear modulus maps are represented in a color-coded elastogram displaying shear-wave velocities in meters per second or tissue elasticity in kilopascals. For the color elastograms, red is usually defined as encoding hard consistency, blue indicates soft consistency, and green and yellow encode intermediate stiffness. When comparing compression sonoelastography with ShWE, it is worth noting that ShWE is regarded as being more objective and reproducible. It offers the advantage of directly assessing tissue elasticity and obtaining quantitative measurements without the requirement for traditional compression [30].

An inherent constraint of ShWE is its limited depth of incursion. Shallow depths can be addressed by the application of a 5-mm layer of coupling ultrasonic gel. Some scanners additionally impose restrictions on the form and size of the ROI for post-analysis purposes. Many scanners necessitate a time delay of a few seconds before the subsequent acquisition, therefore impeding the ability to capture real-time dynamic images of moving structures. Furthermore, ShWE is reactive to the pressure and angle of the transducer, and the shear modulus is influenced by the orientation of the probe in relation to the structures being studied. Techniques for shear wave imaging track how shear waves move through tissue. Unlike compressive or ultrasonic waves, which move in the same direction, shear waves spread out in a tissue displacement vector perpendicular to the tissue displacement direction [31].

ShWE is a type of dynamic elastography that relies on measuring the distribution of tissue propagation velocity in a directionally directed manner. Shear waves are produced by the pulse emission of an ultrasonic machine. Moreover, along with using elastograms for qualitative depiction, ShWE can also measure the absolute value of elasticity of the imaged structure. The speed at which shear waves propagate through tougher tissue is higher. Numerical measurements can be expressed as the shear wave speed in meters per second (m/sec) or as the shear-based analysis of tissue elasticity to determine the modulus in kilopascals (kPa). The key benefits of this approach are minimal reliance on the operator, excellent replicability, and the ability to quantitatively assess the tested parameters [32].

Clinical applications of USE in TMDs

According to Arda et al. [33], the TMJ mechanics and the clinical symptoms that patients report are crucial for the diagnosis of TMD, but they only provide a portion of the disease's overall picture. Innovative methods for assessing the state of the masticatory muscles could help with diagnosis and allow for accurate treatment monitoring. This led to the use of elastography in the evaluation of TMDs. Masseter muscle elasticity index (MEI) and elasticity index are used for strain sonoelastography and real-time sonographic elastography. The results of ShWE are presented in two units, m/s and kPa. The following sections will discuss the various studies performed using both strain and ShWE for TMDs.

Assessing the Normal Values of Stiffness of Disc and Masticatory Muscles in Healthy Adults and Children

Since the introduction of elastography by Ophir et al. in the 1990s, ultrasound elastography has been used in the evaluation of masticatory muscles. In a study including 14 healthy adults, Ariji et al. [34] found that the mean MEI values for the left and right masseter muscles were, respectively, 0.74 ± 0.35 and 0.85 ± 0.44, with a p-value of less than 0.001 and a correlation value of 0.67. The mean MEI values of 10 healthy volunteers in a different study by Ariji et al. [35] were 1.43 ± 0.30. To investigate the MEI values of 10 healthy people, Gotoh et al. [36] conducted a study. The MEI values were found to be 0.84 ± 0.21 and 0.85 ± 0.21 before exercise. The MEI values on the right and left sides were 1.71 ± 0.43 and 1.75 ± 0.43, respectively, when the activity was finished. Koruyucu and Aşantoğrol [37] used ShWE to prospectively study 160 healthy people between the ages of 18 and 59 to determine the reference values of the masseter and temporal muscle hardness values. The masseter muscle's resting hardness was measured transversally at 6.91 kPa and longitudinally at 8.49 kPa; in contrast, the contraction-specific hardness (cSWE) was measured at 31.40 and 35.65 kPa, respectively. At rest and during contraction, the median temporal muscle hardness values were 8.84 and 20.43 kPa, respectively. Male subjects had considerably higher masseter and temporal muscle hardness values during both rest and contraction as compared to their female counterparts.

Öztürk et al. [38] conducted a study to assess the normal values of stiffness of TMJ discs and muscles by ShWE in healthy children and adults. In their prospective study, they evaluated 123 discs and masseter muscle. All individuals' median stiffness disc values were: 23.2 kPa and 2.77 m/s for the posterior sections; 29.10 kPa and 3.07 m/s for the center; and 28.7 kPa (elasticity) and 3.07 m/s (velocity) for the anterior. In comparison to other body parts, posterior stiffness was considerably lower in all participants and age groups. The muscles' average stiffness values were 2.33 ± 1.2 m/s and 16.96 ± 9.01 kPa in closed mouth position, and 28.7 ± 10.2 kPa and 3.23 ± 1.32 m/s in open mouth position. Compared to the anterior or middle regions, the posterior TMJ disc had substantially less rigidity. In comparison to the closed mouth position, the mean stiffness was noticeably higher when the mouth was open. Age, height, weight, or BMI did not correlate with either elasticity or velocity.

Habibi et al. [39] also conducted a study to assess normative values of TMJ disc and masseter muscle. Their study also concluded that the stiffness values exhibited a statistically significant decrease in the posterior region of the disc when compared to the remaining portions in both genders. However, the disc stiffness was higher in females in comparison to males. Regarding age, gender, and mouth position, there were no statistically significant differences seen in the masseter muscle's average stiffness assessments. By enabling the morphological and functional assessments of these muscles and defining ranges for reference parameters, the reference values will facilitate the assessment of pain in the masseter and temporal muscles as well as the diagnosis and prognosis of masticatory muscle disorders.

Assessing the Stiffness of Masseter Muscles in Conditions of Myofascial Pain in TMDs

The application of ultrasound elastography in TMDs has been investigated by various researchers worldwide on myofascial pain. According to Poluha et al. [11], myofascial pain is characterized by the presence of pain or taut bands in one of the muscles of mastication most commonly masseter. Myofascial pain is diagnosed by palpation. The presence of tenderness on palpation of the taut/trigger bands is the hallmark sign of this condition. But this examination is subjective. The trigger bands are active or latent. The latent bands may be clinically not detectable. Many times the trigger bands may not be detected, and this leads to improper diagnosis and incomplete treatment. Brandenburg et al. [40] emphasized that ultrasound elastography is more sensitive than conventional ultrasound at identifying microenvironmental alterations in diseased tissues and elevated resting strain myofascial pain. Several studies have been conducted in detecting muscle stiffness in masseter muscle in patients with TMDs. Both strain wave and ShWE have been used to detect muscle stiffness in these patients.

In strain wave elastography, the MEI has been used to determine muscle stiffness. Ariji et al. [34] conducted a study to assess the MEI of the masseter in eight TMD patients. The MEI ratio was 1.13 ± 0.43 to 11.60 ± 1.19 in symptomatic patients and 0.77 ± 0.31 in healthy patients. Takashima et al. [41] conducted a study among 26 female patients with masticatory myofascial pain (Groups Ia and Ib) and 24 healthy participants. The stiffness of the masseter muscle was assessed by ShWE. Groups Ia and Ib (1.96 m/s (12.5 kPa)) had considerably higher velocity per second than the control group (1.27 m/s (5.25 kPa)) (p < 0.05). The stiffness of the masseter muscle showed a positive correlation with pain intensity (p < 0.05). Females' masseter muscle stiffness, as determined by ShWE, was around two times higher in groups Ia and Ib than in the group of healthy controls. The authors concluded that for measuring the stiffness of the masticatory muscles, ShWE is a valuable tool. According to the various studies stated above, values less than 1.5 have been observed in healthy patients and greater than 1.5 have been observed in patients with myofascial pain.

A Diagnostic Tool to Assess the Decrease in Muscle Stiffness Before and After Treatment in Patients With Myofascial Pain

Monitoring of treatment of TMDs is traditionally done by physical examination and is subjective in nature. ultrasound elastography allows the assessment of stiffness of masseter muscle before and after treatment with high precision and is corroborated by many studies. Olchowy et al. [42] conducted a study to assess masseter stiffness in patients undergoing conservative therapy for masticatory muscle disorders. This prospective cohort study was conducted on 35 patients with masticatory muscle disorders. ShWE was performed both pre- and post-treatment. The muscle stiffness was reduced by 4.1 kPa pre- and post-treatment (12.17 ± 1.17 pre-treatment and 7.96 ± 1.70 post-treatment). The muscle stiffness values correlated with the decrease in VAS scores p < 0.0001.

A Potential Tool to Assess Patients With Bruxism and as a Follow-Up Modality After Treatment in These Patients

Toker et al. [43] conducted a study to evaluate the usefulness of ShWE in the context of bruxism. The left and right masseter muscles were subjected to ShWE under three different conditions: relaxed jaw, 50% of the subject's perceived maximum biting force, and maximally expanded jaw. When the jaw was relaxed, the ShWE was higher in bruxism patients (1.92 m/s ± 0.44) than in the control group (1.66 m/s ± 0.24). The study's conclusions suggest that the use of ShWE for bruxism is feasible and has the potential to be used for the condition's monitoring and diagnosis.

Comparison of ultrasound elastography with MRI elastography

MRI elastography (MRE) is another imaging modality that can be used for assessing muscle stiffness. According to a review by Manduca et al. [44], MRE is an MRI technique that uses phase contrast to visualize mechanical waves and analyze the data to produce precise images that show material properties, namely the shear modulus. Palpation is a subjective method that can solely be employed to assess the regions of the body that are easily reachable. MRE is a quantitative, non-invasive tool analogous to palpation. MRE has been used extensively in liver fibrosis and has not been evaluated in TMDs. The disadvantage of MRE is that it is costly, not widely available, and is technique-sensitive.

Limitations and future directions

Strain wave elastography is subjective and is a semi-quantitative measure of stiffness. The advantages of ShWE over strain wave elastography lie in its capacity to quantitatively evaluate muscle and disc stiffness while simultaneously reducing the impact of operator variability. Large-scale randomized studies should be conducted to assess the effectiveness of ultrasound elastography in the diagnosis and management of TMDs. A comparative assessment of ShWE and MRE should be conducted to understand the mechanical properties of masticatory muscles, discs, and other associated structures of the TMJ.

## Conclusions

Since its inception, ultrasound elastography has been versatilely applied to several systemic diseases. This technique has mostly been employed in the maxillofacial area to evaluate the rigidity of masticatory muscles in individuals with TMDs. Strain wave elastography is a subjective experimental technique with inherent limitations. The objectivity and non-invasive characteristics of shear wave elastography make it highly suitable for extensive application in clinical practice for the diagnosis and monitoring of treatment outcomes in masticatory muscle disorders. It is advisable to undertake additional studies using larger groups of patients and longer periods of follow-up. Ultrasound elastography is a highly promising non-invasive imaging technique. The tool should be used for objective evaluation of patients diagnosed with TMDs. Moreover, it can be utilized after treatment to evaluate the efficacy of the therapy.
